# The role of continuous renal replacement therapy in critically ill children with cancer and multiple organ dysfunction syndrome

**DOI:** 10.1016/j.jped.2025.101473

**Published:** 2025-11-20

**Authors:** Fernanda P.L.F. Veiga, Orlei R. Araujo, Alessandra S. Araujo, Marcela S. Bicalho, Maria C. Andrade, Dafne C.B. Silva

**Affiliations:** a Universidade Federal de São Paulo (UNIFESP), Departamento de Pediatria, Divisão de Nefrologia Pediátrica, São Paulo, SP, Brazil; b Universidade Federal de São Paulo (UNIFESP), Instituto de Oncologia Pediátrica, Cuidados Intensivos Pediátricos - Grupo de Apoio ao Adolescente e a Criança com Câncer (GRAACC), São Paulo, SP, Brazil

**Keywords:** Continuous renal replacement therapy, Multiple organ failure, Critical Care Outcomes, Cancer, Child

## Abstract

**Objectives:**

To describe the demographic and clinical characteristics of pediatric cancer patients receiving continuous renal replacement therapy (CRRT) and to analyze its effect on biochemical markers.

**Methods:**

The authors conducted a cohort study of patients with multiple organ dysfunction syndrome (MODS) who received CRRT in a pediatric oncology intensive care unit. Biochemical measurements were compared at CRRT initiation (D0), after 72 h (D3), and during the final 72 h of therapy.

**Results:**

Fifty-nine cases were analyzed. Hospital mortality was 67.7 %, and the median duration of CRRT was 9 days. Fluid overload was present in 51 % of patients; the mean KDIGO score was 2 (SD: 1). Mechanical ventilation was required in 79.7 %. Among survivors, significant improvements were observed in pH (mean 7.34 on D0 vs. 7.41 on D3; *p* = 0.012; effect size: 0.72) and bicarbonate (mean 22.17 on D0 vs. 27.1 on D3; *p* = 0.003; effect size: 0.77). In non-survivors, lactate levels increased over time (mean 2.3 on D0 vs. 5.37 on the last day; *p* = 0.03; effect size: 0.49). Generalized estimating equation models identified bicarbonate (OR: 0.05), pH (OR: 0.92), PCO₂ (OR: 176), and lactate (OR: 9.8) as significant predictors of in-hospital death. Logistic regression showed that mechanical ventilation (OR: 8.48) and fluid overload in the final 72 h (OR: 1.15) were associated with mortality.

**Conclusions:**

While CRRT’s impact on mortality in MODS remains uncertain, it improved biochemical markers. The findings suggest that metabolic and lactic acidosis, fluid overload, and mechanical ventilation may be modifiable targets to reduce mortality.

## Introduction

Multiple organ dysfunction syndrome (MODS) in children can arise from various triggers during critical illness. It is defined by the concurrent failure or dysfunction of multiple organ systems, including the respiratory, cardiovascular, renal, hematologic, and hepatic systems. MODS typically reflects a severe, dysregulated systemic inflammatory response, often precipitated by conditions such as infection or shock [1]. Children with cancer are particularly vulnerable, as chemotherapy-induced organ toxicity may predispose them to further dysfunction in the presence of infection or inflammation [2].

Acute kidney injury within the context of MODS is a particularly grave development, and early initiation of renal replacement therapy is often vital to improving survival outcomes. Continuous renal replacement therapy (CRRT)—delivered via venovenous hemodialysis, venovenous hemofiltration, or venovenous hemodiafiltration—has become the predominant modality of renal support in critically ill pediatric patients [3]. CRRT is especially favored in hemodynamically unstable children with fluid overload (FO) due to its capacity to deliver controlled ultrafiltration rates and simulate continuous renal function. Nonetheless, the requirement for CRRT is frequently associated with a poor prognosis [4].

This study aimed to describe the demographic and clinical characteristics of pediatric cancer patients receiving continuous renal replacement therapy and to analyze its effect on biochemical markers, such as pH, electrolytes, and nitrogenous end products of metabolism (urea and creatinine), in addition to trying to identify potentially modifiable variables that may interfere with the prognosis of critically ill children with cancer.

## Methods

This retrospective, single-center study was conducted at a hospital exclusively dedicated to treating pediatric cancer. The study protocol was approved by the Research Ethics Committee of the Federal University of São Paulo (certificate of ethical appraisal-CAAE No 36,144,820,700,005,505). Informed consent was waived because of the nature of the study. Patients aged up to 18 years, diagnosed with cancer and at least two organ dysfunctions according to the criteria of the Pediatric Organ Dysfunction Information Update Mandate (PODIUM) expert panel [5], and who underwent CRRT in a six-year period (2013 to 2019) were included in the study. Demographic data, oncological diagnoses, laboratory results of blood gas analysis, electrolytes, complete blood count, coagulation tests, renal and liver function, need for mechanical ventilation, and vasoactive drugs with VIS score [6], and outcome of death during hospitalization or survival were collected from the medical records. The percentage of fluid overload was defined using the method described by Goldstein et al.: ([fluid input – fluid output]/[intensive care unit admission weight] × 100) [7]. The authors assessed the percentage of fluid overload in the 72 h before the start of CRRT, in the 72 h after the start, and in the final 72 h. The severity of acute kidney injury was quantified using the KDIGO-AKI score [8]. The pediatric intensivist and nephrologist teams jointly performed CRRT prescription and management, and all patients received continuous venovenous hemodiafiltration. Prismaflex® dialysis machines (Gambro - Baxter) were used for CRRT in all patients. Citrate anticoagulation was used in 83 % of patients. The dialysate composition followed the standard at 0.61 % (sodium 105 mEq/L and magnesium 1.5 mEq/L), supplementing up to 140 mEq/L of sodium with 20 % sodium chloride or bicarbonate. Dialysis/replacement flow is standardized in the PICU at 35 to 50 mL/kg/hour. As mortality reported in the literature increases with fluid overload percentages > 10 %, the authors generally evaluate starting CRRT with percentages between 5 % and 10 %, with no response to diuretics, or with worsening renal function or signs of pulmonary edema. Initiation is mandatory if it is above 10 %.

Descriptive statistics encompassed measures of central tendency and dispersion, including means, standard deviations (SD), medians, and interquartile ranges (IQR; 25th–75th percentiles), as well as absolute and relative frequencies. Differences in proportions were assessed using the chi-square test. For repeated measures analysis, the Friedman test was employed, followed by post hoc comparisons using the Durbin test. To quantify the magnitude of observed effects, the authors applied the rank-biserial correlation [9], interpreted as follows: a small effect (< 0.3), a medium effect (0.3–0.5), and a large effect (> 0.5). To model the relationship between repeated measures and the outcome of in-hospital death, the authors used generalized estimating equations (GEE) with an autoregressive (AR-1) correlation structure. This approach accounts for within-subject correlations and is appropriate for both normal and non-normal longitudinal data. Like the generalized linear mixed model, GEE involves solving a system of equations to estimate model parameters (i.e., coefficients) without relying on strict parametric assumptions [10]. The marginal means of the variables for the outcome were obtained. Logistic regression (bivariate and multivariate) was used to evaluate the following variables as risk factors for the outcome of in-hospital death: mechanical ventilation, estimated renal clearance (Schwartz), KDIGO-AKI score, diagnosis of leukemia or relapse of oncological disease, need for vasoactive drugs, hematopoietic stem cell transplantation (HSCT), accumulated fluid balance values, and age.

Analyses were performed using the R environment (R: A language and environment for statistical computing. R Foundation for Statistical Computing), and Jamovi software version 2.6.

## Results

Data from 59 patients were analyzed. Table 1 presents the demographic and other characteristics. The main reasons for admission to the PICU were sepsis (12, 20.3 %), acute respiratory failure (24, 40.6 %), acute renal failure (6, 10.1 %), postoperative (6, 10.1 %), and electrolyte disturbances (6, 10.1 %). Acute respiratory failure was associated with fluid overload of > 10 % in 15 (62.5 %) of 24 cases. Seventeen patients (29 %) had hyperlactatemia > 2.2 mmol/L at the start of CRRT; of these, 12 had a diagnosis of septic shock requiring vasoactive drugs, 1 had a diagnosis of sepsis, and 10 had liver dysfunction.

**Table 1:** General data.

Age (years, median, IQR)	10.1	3.9–13.7	
Female sex	26.0	44.1 %	
Fluid overload > 10 % in the 72 h prior to CRRT	26	44 %	
Electrolyte disturbances	25.0	42.4 %	
Multiple organ dysfunction (median number of systems involved, IQR)	3	3–4	
Hepatic dysfunction	36	61.0 %	
Hematologic dysfunction	29	49.2 %	
Coagulation system dysfunction	10	16.9 %	
Respiratory dysfunction	47	79.7 %	
Cardiovascular dysfunction	26	44.1 %	
Renal dysfunction	59	100 %	
Need for vasoactive drugs	35	59.3 %	
VIS score in patients requiring vasoactive drugs (mean, SD)	29.5	56.8	
Cancer diagnoses			
Acute lymphoblastic leukemia	10	16.9 %	
Acute myelocytic leukemia	5	8.5 %	
Other leukemias	8	13.6 %	
Hodgkin lymphoma	1	1.7 %	
Non-Hodgkin lymphoma	4	6.8 %	
Central nervous system tumors	6	10.2 %	
Neuroblastoma	2	3.4 %	
Retinoblastoma	2	3.4 %	
Wilms tumor	2	3.4 %	
Others	19	32.2 %	
Hematopoietic stem cell transplantation (HSTC)	18	30.5 %	
Mechanical ventilation	47	79.7 %	
Peak pressure (start of CRRT, cmH_2_O, mean, SD)	23	8.7	
FiO2	54 %	25 %	
PEEP (start of CRRT, cmH2O, mean, SD)	9.7	2.5	
SpO2/FiO2 ratio (start of CRRT, mean, SD)	216	97.8	
Duration of CRRT (days, median, IQR)	9	4.5–15	
AKI stage according to KDIGO on the CRRT starting day (median, IQR)	2	1–3	
Deaths during hospitalization	40	67.7 %	
Length of hospitalization (days, median, IQR)	27	15–45	

Data relating to laboratory tests at the three assessment times (start of renal support therapy, on the third day, and on the last day) are shown in Table 2.

**Table 2:** Laboratory tests on the day CRRT started, on the 3rd day after and on the last day (median of 9 days). Differences assessed by the Wilcoxon signed-rank test, with the sizes of the effects by rank-biserial correlation, when significant.

	Start of CRRT (D0)	After 72 h of CRRT (D3)			Last 72 h of CRRT (End)		
	Mean (SD)	Mean (SD)	p-value (D3 - D0)	Effect size	Mean (SD)	p-value (End - D0)	Effect size
Creatinine (mg/dL)	1.6 (1.2)	0.9 (0.5)	<0.001	−0.74	0.8 (0.6)	<0.001	−0.78
Urea (mg/dL)	89.3 (68.9)	51.9 (38.3)	<0.001	−0.63	53 (28.7)	<0.001	−0.59
pH	7.3 (0.1)	7.4 (0.1)	0.003	0.46	7.3 (0.2)	0.62	-
PCO_2_ (mmHg)	44.4 (12.6)	46.4 (19.9)	0.6	-	44.7 (17.3)	0.7	-
Base Excess (mmol/L)	−5.1 (6.8)	−2.4 (18.7)	<0.001	0.51	−6.6 (18.2)	0.5	-
PO_2_ (mmHg)	118.8 (275.9)	89 (32.7)	0.5	-	77.9 (37.1)	0.25	-
Bicarbonate (mmol/L)	20.9 (5.7)	24.4 (6.1)	<0.001	0.51	22.6 (4.6)	0.2	-
Lactate (mmol/L)	2.4 (2.4)	2.8 (4.5)	0.43	-	4 (5)	0.15	-
Albumin (g/dL)	3.2 (0.6)	3.5 (0.7)	0.02	0.46	3.5 (0.8)	0.13	-
Sodium (mmol/L)	142.6 (8.2)	141.2 (7.8)	0.2	-	140.1 (7.1)	0.1	-
Potassium (mmol/L)	4.2 (0.9)	3.9 (0.6)	0.05	-	4.2 (0.8)	0.78	-
Calcium (mmol/L)	1.15 (0.14)	1.18 (0.17)	0.19	-	1.25 (0.2)	0.16	-
Magnesium (mg/dL)	2 (0.5)	2 (0.4)	0.7	-	1.9 (0.4)	0.15	-
Phosphorus (mg/dL)	5.5 (2.9)	3.8 (1.8)	<0.001	−0.54	4 (1.4)	0.002	−0.53
Hemoglobin (g/dL)	8.9 (1.5)	9.4 (1.4)	0.1	-	9.4 (1.6)	0.02	-
Leukocytes (cells/ µL)	7218.6 (9041.8)	9921.8 (25,180)	0.36	-	6040.9 (7751)	0.45	-
Platelets (cells/ µL)	85,290.7 (109,558)	58,656.3 (75,116)	0.08	-	52,664.4 (50,132)	0.1	-
C-reactive protein (mg/dL)	164.3 (127.8)	137.1 (113.2)	0.5	-	156.8 (124.2)	0.95	-
Prothrombin time INR	1.6 (0.4)	2.1 (3.3)	0.5	-	1.3 (0.3)	0.23	-
Partial Thromboplastin time INR	1.5 (0.4)	2.5 (7.3)	0.4	-	2.5 (6.4)	0.24	-
Oxaloacetic transaminase (U/L)	184.9 (316.3)	304.1 (521.4)	0.4	-	213 (545)	0.8	-
Pyruvic Transaminase (U/L)	100.7 (156.6)	158.6 (405.5)	0.9	-	68.7 (94.7)	0.6	-

**Figure 1:**
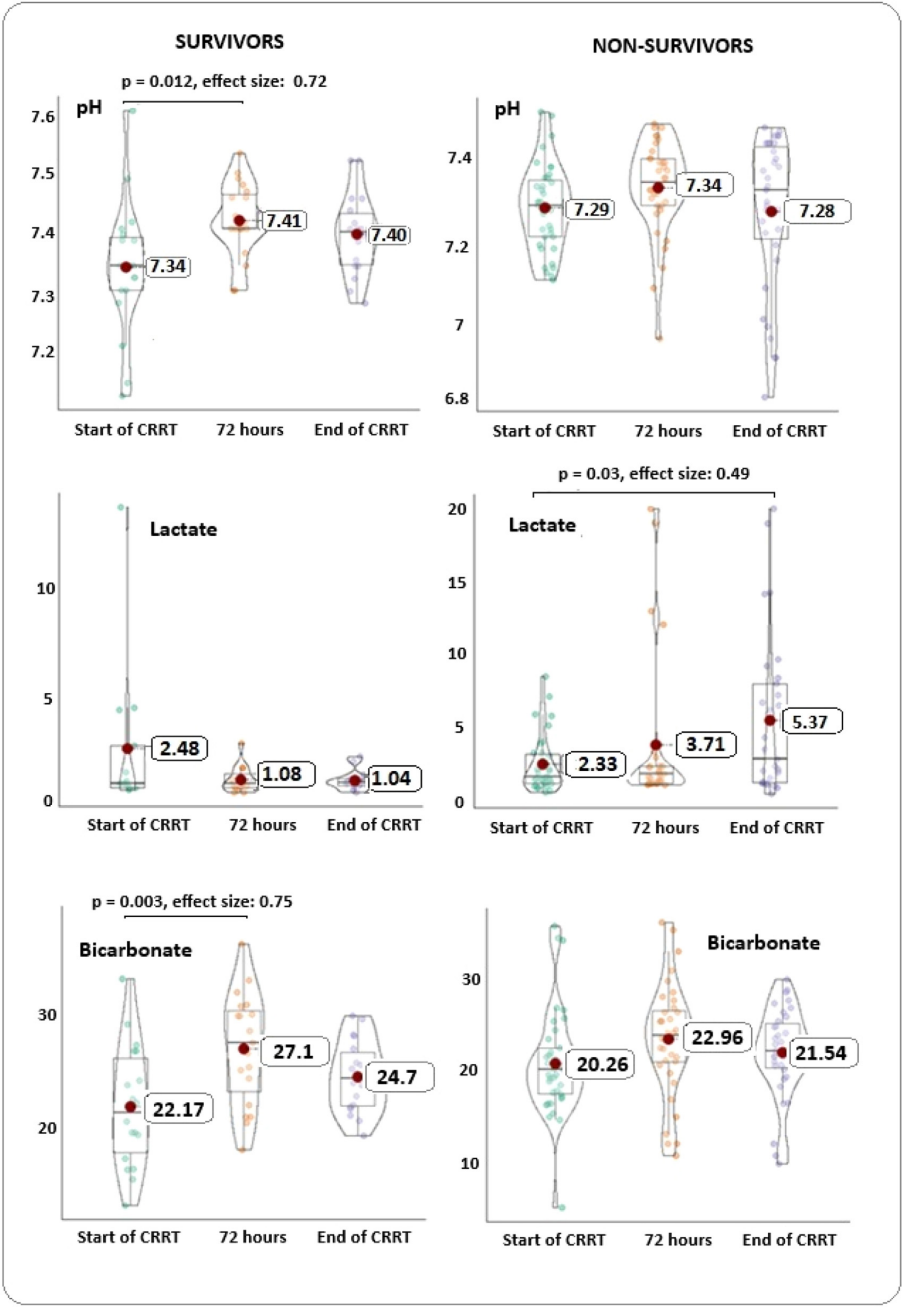
Violin boxplots showing the differences between pH, lactate and bicarbonate measurements at the beginning of CRRT, after 72 h and on the last day of support, for survivors and deceased. The red dots symbolize the means. In survivors, a large effect was observed with significant differences between the beginning and after 72 h of dialysis, for pH and bicarbonate. The differences were not significant in patients who died. Lactate showed a downward trend in survivors and a significant upward trend in those who died, throughout the three measurements.

In the models of the generalized estimating equations for the outcome “in-hospital death”, the β-coefficients and estimated marginal means were not significant for hemoglobin, leukocytes, platelets, transaminases, albumin, sodium, potassium, calcium, phosphorus, magnesium, base excess, urea, and creatinine. Table 3 shows the models for pH, partial pressure of carbon dioxide (PCO_2_), bicarbonate, and lactate. A temporal evolution for pH, lactate, and bicarbonate in the survivors and non-survivors is shown in Figure 1. In survivors, pH and bicarbonate increased significantly between D0 and D3 of CRRT (for pH, mean of 7.34 (SD: 0.11) on D0 to 7.41 (SD: 0.06) on D3 (*p* = 0.012, effect size 0.72); for bicarbonate, mean of 22.17 (SD: 5.13) on D0 to 27.1 (SD: 4.75) on D3 (*p* = 0.003, effect size 0.77). In non-survivors, lactate levels during CRRT increased from 2.3 (SD: 1.9) on D0 to 5.37 (SD: 5.5) on the last day (*p* = 0.03, effect size = 0.49).

**Table 3:** Models generated by generalized estimating equations (GEE) and multivariate logistic regression.

**GEE**	Coefficient (β)	Standard error	Odds ratio	95 % CI of OR	Estimated marginal mean - deceased	Estimated marginal mean - survivors	p-value
Bicarbonate	−3.11	0.83	0.05	0.008 – 0.22	21.6	24.7	<0.001
pH	−0.08	0.01	0.92	0.89 – 0.95	7.30	7.38	<0.001
PCO_2_	5.17	2.11	176	18.6 - 46,200	46	40.7	0.015
Lactate	2.28	0.61	9.8	2.98 – 32.5	3.73	1.45	<0.001
**Logistic models**							
Mechanical ventilation	2.14	0.83	8.48	1.67 - 43	-	-	0.010
Percentage of fluid overload in the final 72 h	0.14	0.06	1.15	1.03 – 1.28	-	-	0.012

The bivariate logistic models showed significance for mechanical ventilation and fluid balance accumulated in the final 72 h. These two variables were independent in-hospital mortality predictors (Table 3). The SpO2/FiO2 ratio was not a predictor of the outcome and was not useful for adjusting the model.

Both survivors and non-survivors demonstrated a significant reduction in fluid overload during CRRT. Among non-survivors, the cumulative FO percentage in the 72 h preceding CRRT averaged 9.9 % (SD: 7.3 %), decreasing to −1.15 % (SD: 9.8 %) after 72 h, and to 1.26 % (SD: 7.6 %) in the final 72 h. The effect size for the comparison between FO in the 72 h before and after CRRT was −0.76 (*p* < 0.001), and −0.72 (*p* < 0.001) when comparing the final 72 h with the pre-CRRT period.

**Figure 2:**
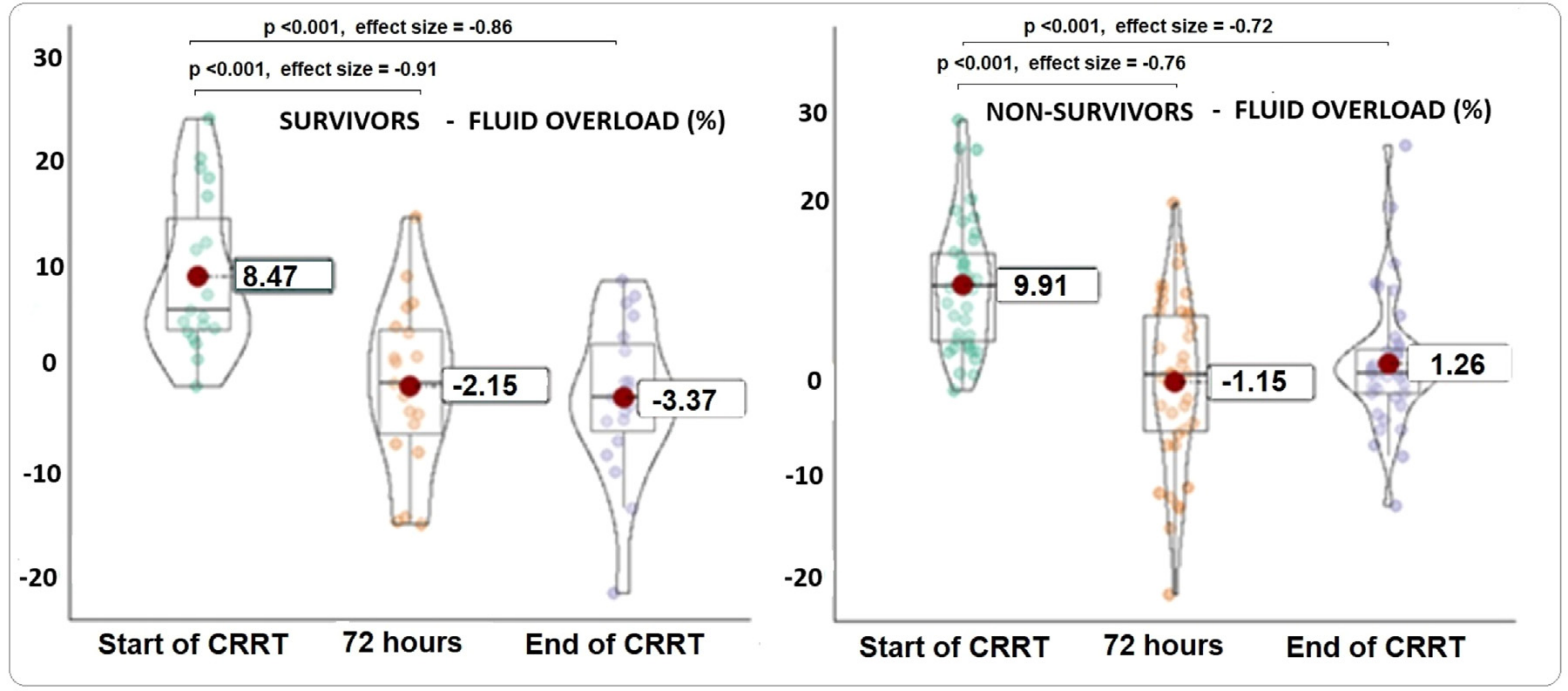
Violin boxplots, showing the variations in the percentages of fluid overload in 72 h, in the three instances evaluated.

In survivors, the cumulative mean FO in the 72 h prior to CRRT was 8.47 % (SD: 7.6 %), which decreased to −2.15 % (SD: 8.1 %) after 72 h, and to −3.37 % (SD: 7.5 %) in the final 72 h. The effect size for the comparison between the pre- and post-CRRT 72-hour periods was −0.91 (*p* < 0.001), and −0.86 (*p* < 0.001) when comparing the pre-CRRT and final 72-hour values. Figure 2 illustrates the variations in fluid overload percentages over time.

All patients received furosemide in doses of up to 6 mg/kg/day, but a negative water balance was only obtained in 2 patients (3.3 %) in the 72 h before CRRT.

In the subgroup of patients undergoing HSCT (*N* = 18), there were 12 deaths (67 %), with no statistical difference for the other patients (*N* = 41, 28 deaths (68.2 %, *p* = 0.9). In the subgroup of patients with KDIGO 3, the mortality rate was 64 % (16/25), similar to that observed in the KDIGO 2 subgroup (12/17, 70.5 %, *p* = 0.65)

Among the survivors, only one patient (5.2 %) with Wilms' tumor remained in need of hemodialysis after hospital discharge.

## Discussion

In this cohort of critically ill children with multiple organ dysfunction syndrome, CRRT led to rapid improvements in fluid balance, metabolic acidosis, electrolyte disturbances, and nitrogenous waste levels, with the most pronounced effects observed within the first 72 h. Despite these biochemical improvements, the in-hospital mortality rate was notably high at 67 %. The literature on this topic remains limited; however, Miklaszewska et al. reported a mortality rate of 74 % among patients with MODS requiring CRRT [11]. Similarly, Cortina et al. found a mortality rate of 77.8 % in patients with oncohematological diseases undergoing CRRT [12]. Thadani et al. described a significant decline in renal recovery following 30 days of CRRT, with only 40 % of patients achieving recovery by approximately 90 days. Their study also indicated that hematologic dysfunction and cancer were associated with increased odds of major renal adverse events, and that progression of organ dysfunction correlated with failure to recover renal function [13]. In the present sample, 49 % of patients presented with hematologic dysfunctions, including leukopenia and thrombocytopenia. All patients presented with multiple organ dysfunction syndrome (MODS) at the initiation of CRRT, with a median involvement of three organ systems, contributing to a particularly poor prognosis. Ma et al. reported an ICU mortality rate of 41.3 % among pediatric patients with cancer, sepsis, and CRRT requirement; however, their analysis did not account for the concurrent presence of additional organ dysfunctions [14]. A similar pattern was observed in the study by Raymakers-Janssen et al., which reported an ICU mortality rate of 54.4 % among cancer patients undergoing hematopoietic stem cell transplantation and receiving CRRT [15]. MODS is characterized by a multifactorial pathophysiology, primarily driven by tissue hypoxia and systemic inflammatory responses. As dysfunction develops concurrently across multiple organ systems, it is likely that similar pathological mechanisms are at play in each. Hypoxia contributes to cellular injury and death, resulting in necrotic areas within the affected organs [16]. Reversible dysfunction induced by inflammation may serve as a protective mechanism to prevent irreversible organ “death” resulting from hypoxia. Although this short-term adaptation allows individual organs the possibility of recovery, it can lead to systemic dysregulation and ultimately death. During the inflammatory response, elevated levels of phospholipases, reactive oxygen species (ROS), and reactive nitrogen species (RNS) contribute to cell membrane damage. These mediators can simultaneously affect multiple organs, resulting in profound and widespread dysfunction [17].

The results of generalized estimating equation models are noteworthy, demonstrating that patients who survived had higher marginal means of bicarbonate and pH, and lower marginal means of lactate and PCO₂, compared to those who died. Acute hypercapnic acidosis is generally well tolerated in patients with acute lung injury; however, robust neuroendocrine responses are required to counteract the physiological effects of elevated PCO₂, such as negative inotropy and vasodilation. Additionally, mechanisms responsible for maintaining intracellular pH must remain functional—particularly in vital organs like the heart and brain[14] — which may be compromised in critically ill patients. Hypercapnia can cause alveolar epithelial damage, reducing alveolar fluid clearance [18]. CRRT may be effective in removing CO₂ when a dedicated removal cartridge is coupled to the hemofilter. However, this technique was not utilized during the study period due to a lack of availability. Even today, such cartridges are only approved for use in patients weighing over 30 kg, limiting their applicability in smaller pediatric populations. Theoretically, given the observed correlation between metabolic acidosis and elevated PCO₂ with mortality in the present study, adjustments to CRRT aimed at correcting acidosis and facilitating CO₂ removal may help reduce mortality in patients with similar clinical profiles. Renal replacement therapy is effective in managing metabolic acidosis. However, in intermittent hemodialysis, the rapid bicarbonate flux from the dialysate to the patient can lead to excess CO₂ generation and subsequent alkalosis. This phenomenon is less common in CRRT due to the slower flux and delivery rate of the buffer solution [19].

The use of citrate for anticoagulation provides a continuous source of bicarbonate (or its equivalent, citrate), which helps reduce the prevalence and severity of hyperchloremic acidosis in the absence of tissue hypoxia-ischemia. Regional citrate anticoagulation is commonly associated with elevated bicarbonate levels and metabolic alkalosis. However, inadequate citrate metabolism—such as in cases of severe liver failure or septic/cardiogenic shock with tissue hypoperfusion—can lead to metabolic acidosis [20]. Lactic acidosis remains relatively common [19]. Elevated lactate levels have been linked to increased mortality across various critical illness settings, particularly in septic shock [21]. During tissue hypoxia, oxidative phosphorylation, ATP synthesis, and the reoxidation of nicotinamide adenine dinucleotide are inhibited. This leads to the conversion of pyruvate to lactate and inefficient ATP production, resulting in hyperlactatemia. Interestingly, in sepsis, lactic acidosis may occur even in the absence of tissue hypoxia and with normal cellular ATP levels, suggesting that lactate accumulation may stem from dysregulated cellular metabolism [22]. Treatment of lactic acidosis focuses on restoring adequate tissue perfusion. Continuous renal replacement therapy with bicarbonate-containing solutions can help manage acidemia until the underlying cause is resolved [23]. In the present observations, patients who survived showed a downward trend in lactate levels, indicating a possible effect of CRRT on lactate clearance. Conversely, patients who died exhibited an upward trend, likely reflecting worsening organ function. Failure to clear lactate was associated with a worse prognosis in this study.

Consistent with the findings of Thadani et al. [13], the authors observed that respiratory failure was the most common dysfunction at the initiation of CRRT. In the present study, respiratory dysfunction was frequently associated with fluid overload, and mechanical ventilation emerged as an independent predictor of mortality when analyzed alongside the percentage of fluid overload during the final 72 h of CRRT. Specifically, the need for mechanical ventilation increased the risk of death by 8.5 times, and each percentage point increase in fluid overload raised the risk by 15 %. In the study by Pechlaner et al. [24], children with MODS had an admission mortality rate of 50.0 %, and mechanical ventilation increased the risk of death by 25.9 times. Mechanical ventilation may exacerbate renal dysfunction through three proposed mechanisms: (1) hypoxemia and hypercapnia impair chemoregulation of renal artery diameter, reducing renal blood flow; (2) ventilation-induced release of inflammatory mediators from the lungs can cause remote injury to renal tubular cells; and (3) hemodynamic changes associated with ventilation may reduce venous return and cardiac output, further compromising renal perfusion [25]. Conversely, fluid overload can contribute to lung injury. One proposed mechanism involves the release of atrial natriuretic peptide (ANP) due to mechanical stress in the atrium caused by volume overload. ANP may damage the endothelial glycocalyx, increasing vascular permeability and leading to tissue edema. Additionally, elevated central venous pressure and capillary hydrostatic pressure can promote pulmonary edema, impairing both ventilation and perfusion [26]. This creates a vicious cycle in which respiratory and renal dysfunctions exacerbate each other, contributing to poor outcomes. However, these conditions are potentially modifiable. Adjustments in ultrafiltration rates and, when necessary, vasoactive drug infusion rates can help manage fluid overload more effectively. Reconciling a ventilation strategy that simultaneously protects both the lungs and kidneys remains a complex challenge. Evidence suggests that low tidal volume ventilation does not reduce the incidence of kidney injury [27], and high PEEP levels may be causally linked to severe renal dysfunction. To minimize morbidity, it is essential to optimize PEEP not only from a respiratory or cardiopulmonary standpoint but also through a lung-heart-kidney integrative approach [25].

This study has several limitations, the most evident being its retrospective design, which relied on the accuracy of medical record documentation. The high number of patients receiving mechanical ventilation and sedation hindered the assessment of neurological function; consequently, this system was excluded from the list of dysfunctions. Additionally, the small sample size from a single center limited the possibility of conducting more extensive subgroup analyses. Therefore, the findings may not be generalizable to other institutions with different patient populations or clinical practices.

Patients with unfavorable outcomes demonstrated poorer control of metabolic and lactic acidosis during CRRT, as well as higher percentages of fluid overload throughout the course of therapy in the context of MODS. Respiratory failure was the most frequently observed dysfunction associated with the initiation of CRRT and emerged as an independent predictor of mortality, alongside the degree of fluid overload at the end of renal replacement therapy. These findings suggest that metabolic and lactic acidosis, fluid overload, and mechanical ventilation represent potentially modifiable variables that could help reduce mortality in critically ill pediatric oncology patients requiring CRRT. Serial lactate measurements — forming a lactate curve — may serve as a valuable prognostic tool.

## Financial support used for the study

Only institutional.

## Authors’ contributions

Conception and design of the study: DCBS, MCA, ORA

Acquisition of data, or analysis and interpretation of data: FPLV, MSB. ASA, ORA

Drafting the article or revising it critically for important intellectual content: ORA, MCA, DCBS

Final approval of the version to be submitted: all authors.

## Data availability statement

The data that support the findings of this study are available from the corresponding author.

## Conflicts of interest

The authors declare no conflicts of interest.
